# Toxicological Legacy of Polycyclic Aromatic Hydrocarbons from a Tire Fire-Urban Soil Contamination and Cancer Risk Assessment

**DOI:** 10.3390/toxics14070543

**Published:** 2026-06-23

**Authors:** Kamil Pająk, Alicja Trawińska, Marcin Łapicz, Andrzej R. Reindl

**Affiliations:** 1 Department of Environmental Toxicology, Faculty of Health Sciences, Medical University of Gdansk, 80-210 Gdansk, Poland; kamil.pajak@gumed.edu.pl (K.P.); alicja.trawinska@gumed.edu.pl (A.T.); 2 Department of Rescue Operations Management, Firefighting and Communication, Faculty of Safety Engineering and Civil Protection, Fire University, 52/54 Slowackiego St., 01-629 Warsaw, Poland; mlapicz@apoz.edu.pl

**Keywords:** waste tire fire, polycyclic aromatic hydrocarbons (PAHs), HYSPLIT dispersion modeling, incremental lifetime cancer risk (ILCR), ecological risk quotient (RQ), soil toxicological assessment

## Abstract

Landfill tire fires are complex environmental disasters generating toxic pollutants with severe health risks. This study quantified emission dynamics and toxicological consequences of a large-scale tire fire in an urban ecosystem. A comprehensive source-to-receptor approach was applied, integrating Hybrid Single-Particle Lagrangian Integrated Trajectory (HYSPLIT) atmospheric dispersion modeling with comparison against air quality monitoring data. Soil samples collected from the fireground and surrounding urban allotment gardens were analyzed for tire-specific tracers (Zn) and 16 priority polycyclic aromatic hydrocarbons (PAHs). Human health risks were assessed using Incremental Lifetime Cancer Risk (ILCR), Toxic Equivalency Quotient (TEQ), and Mutagenic Equivalency Quotient (MEQ) metrics. Fire emissions were dominated by particulate matter (PM_10_: 1.34 t) and PAHs (17.7 kg). Soil at the fire site showed severe contamination (Σ PAHs: 148.9 mg/kg), with benzo[a]pyrene as the primary carcinogen. The cumulative ILCR for children reached 9.7 × 10^−4^, exceeding the commonly used upper regulatory benchmark of 10^−4^. Dermal contact was identified as the dominant exposure pathway for pyrogenic PAHs. Elevated risk levels persisted at distal residential sites (ILCR: 10^−5^–10^−4^), indicating long-term environmental contamination Ecological risk quotients (RQ) exceeded unity for PAHs across all fire-impacted locations and for Zn and Cu in the immediate vicinity of the fire scene. These findings demonstrate that acute tire fire events can evolve into persistent terrestrial health hazards, highlighting the critical role of dermal exposure in PAH uptake and the need for long-term environmental monitoring and adaptive land-use management strategies to mitigate chronic health risks in urban populations.

## 1. Introduction

Industrial fires and waste storage disasters represent critical episodic sources of polycyclic aromatic hydrocarbons (PAHs), and heavy metals, posing a multi-dimensional threat to the biosphere. These events release complex pyrogenic mixtures that lead to acute environmental degradation, particularly in downwind sectors where atmospheric deposition and firefighting runoff intersect [1,2]. Unlike chronic industrial emissions, tire fires are characterized by highly inefficient, oxygen-limited combustion, generating residues that persist in the soil matrix for decades. Integrated planetary health perspectives suggest that such persistent pollutants create secondary exposure loops for local populations through soil-to-crop transfer and direct contact [3].

The global magnitude of waste tire accumulation, estimated at over one billion units generated annually, exacerbates the frequency of these uncontrolled combustion events. Tire rubber is a sophisticated chemical matrix, comprising approximately 50 (wt%) natural and synthetic polymers, 25 (wt%) carbon black, and 10–15 (wt%) metallic reinforcements (predominantly steel and brass) [4,5]. During combustion, especially in the oxygen-limited, smoldering conditions characteristic of large stockpiles, this matrix undergoes incomplete thermal degradation. This leads to the de novo synthesis of high-molecular-weight (HMW) PAHs, many of which exhibit potent carcinogenic and mutagenic properties [6]. Furthermore, the mobilization of tire-specific inorganic markers, such as Zinc (Zn) and Copper (Cu), creates a persistent chemical “fingerprint” that disrupts terrestrial health for decades [3].

The public health significance of such events is profound but frequently underestimated due to a lack of interdisciplinary oversight. Current toxicological frameworks, such as the Toxic Equivalency (TEQ) and Mutagenic Equivalency (MEQ) approaches, are essential for characterizing the risk hazard by these complex mixtures. Specifically, the MEQ provides a critical proxy for the DNA-damage potential of pyrogenic residues, accounting for the genotoxic pathways that lead to long-term health deterioration. However, a major gap exists in the literature regarding the integrated spatio-temporal dynamics of real-world tire fire plumes at the environment–human interface. Most existing studies are limited to either laboratory-scale simulations, which fail to capture the complexity of large-scale fire dynamics, or isolated soil sampling without atmospheric transport context. There is a critical lack of data linking high-resolution emission factors with validated atmospheric dispersion models and in situ multi-pathway risk assessments (ILCR), particularly in vulnerable urban sectors such as allotment gardens where the soil-to-human transfer represents a direct One Health challenge.

The novelty of this study lies in its integrated and holistic “source-to-receptor” analytical framework. We reconstruct a major landfill tire fire event by synthesizing: (i) quantified source-term emissions with rigorous uncertainty propagation, (ii) HYSPLIT-based atmospheric dispersion modeling validated against regional air quality monitoring data, and (iii) a systematic in situ investigation of soil contamination. By applying a dual-tier ecological risk assessment (RQMPC vs. RQNCs) alongside demographic-specific health risk models (ILCR for adults and children), we demonstrate the transition from an acute atmospheric crisis to a long-term terrestrial toxicological legacy. This research provides a robust, evidence-based foundation for public health authorities to implement holistic remediation strategies and safeguard sensitive populations by addressing the interconnected health of the shared environment.

## 2. Materials and Methods

### 2.1. Study Site and Fire Event Characteristics

In Poland, emergency incidents are managed by the National Firefighting and Rescue System. For every intervention, a standardized report is generated within the Decision Support System (SWD). These records include incident categorization, geographical coordinates, facility dimensions, ownership details, and tactical data such as the quantity of extinguishing agents and the number of deployed units [7].

This study investigated the environmental impact of a fire at a waste tire storage facility. The ignition occurred on 13 October 2025, at approximately 02:50 a.m., with the official emergency notification recorded at 02:55 a.m. Suppression operations lasted 9 h and 27 min. During the intervention, approximately 60 m^3^ of water and 1300 L of a synthetic foaming agent (Roteor M Premium, PCC Exol SA, Brzeg Dolny, Poland) were applied. The fire affected an estimated area of 450 m^2^, with a total combustion volume of approximately 450 m^3^.

### 2.2. Soil Sampling

**Figure 1:**
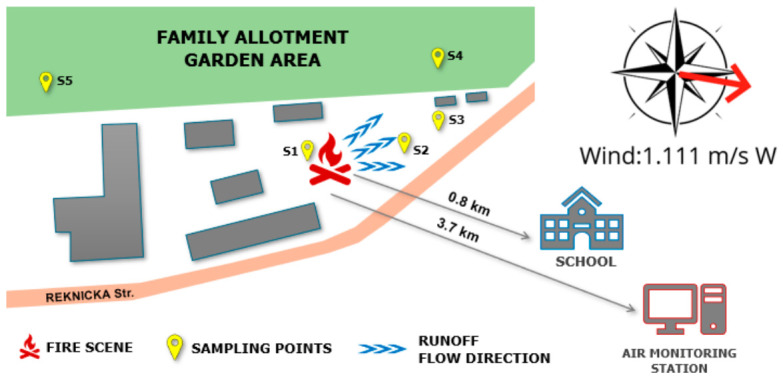
Spatial distribution of soil sampling locations in relation to the fire scene, prevailing wind direction during the fire event, and observed post-fire runoff pathways. Blue arrows indicate dominant surface water flow during firefighting activities. Red arrow indicate wind direction.

Soil sampling was designed to evaluate spatial variability linked to direct combustion, atmospheric deposition, and firefighting runoff. Samples were collected in situ from the upper horizon (0–20 cm) at strategically selected locations. Sampling locations were deliberately stratified based on meteorological parameters and topographical runoff observations: S1 represents the fire origin (ignition point), S2 and S3 capture the immediate downwind and primary firefighting water runoff pathways, S4 was selected to assess human exposure in distal, sensitive residential/agricultural zones (allotment gardens) along the dominant wind vector, and S5 serves as an upwind, unimpacted local background reference (Figure 1). To ensure sample integrity, single-use gloves and decontaminated tools were employed at each point.

The collected material was immediately transferred to airtight string-lock polyethylene bags, wrapped in aluminum foil to prevent photo-degradation of organic analytes, and stored in a portable cooler at 4 °C. This rigorous cold-chain protocol was maintained until laboratory delivery to minimize the volatilization of low-molecular-weight PAHs and biological transformation of the soil matrix.

### 2.3. Emission Factors of Combustion Products

Annual air quality assessments in Poland monitor 12 regulated substances, including SO_2_, NO_2_, CO, C_6_H_6_, O_3_, PM_2.5_, PM_10_, and PM_10_, alongside heavy metals (Pb, As, Cd, Ni) and benzo(a)pyrene. In this study, emission fluxes were quantified for CO_2_, CO, NO_x_, SO_2_, PM_10_, volatile organic compounds (VOCs), polycyclic aromatic hydrocarbons (PAHs), and CH_4_. These pollutants were selected based on their toxicological relevance, climate forcing potential, and the availability of robust emission factors (*EFs*) for uncontrolled combustion. Total emissions (*E_x_*) were estimated following the methodological framework of Bihałowicz et al. [8] adopted from Downard et al. [5] and Lemieux and Ryan [9], expressed as:

(1) $$EM_{\left\{x\right\}}=q\;\cdot V\;\cdot EF_{\left\{x\right\}}$$

where *q* is the bulk density of the waste tires (kg/m^3^), *V* is the estimated volume of consumed tires (m^3^), and *EF_x_*, is the emission factor for pollutant (g/kg). Due to the heterogeneous nature of tire landfill fires, characterized by oxygen-limited regimes and the suppressive effects of water/foam, emissions were temporally averaged assuming a quasi-steady combustion phase.

### 2.4. Uncertainty Analysis

Uncertainty in emission estimates was propagated using the standard Taylor series expansion for multiplicative variables, consistent with IPCC guidelines and Bihałowicz et al. [8]:

(2) $$\frac{u\left({EM}_{x}\right)}{{EM}_{x}}=\sqrt{{\left(\frac{u\left(q\right)}{q}\right)}^{2}+{\left(\frac{u\left(V\right)}{V}\right)}^{2}+{\left(\frac{u\left(EF_{x}\right)}{EF_{x}}\right)}^{2}}$$

where $u(EM_{x})/EM_{x}$ is the relative uncertainty of the estimated emission of pollutant x, u(q)/q is the relative uncertainty associated with the bulk density of waste tires, u(V)/V is the relative uncertainty of the burned volume, and $u(EF_{x})/EF_{x}$ is the relative uncertainty of the literature-derived emission factor for pollutant x. This error-propagation formula is a standard analytical expression and was adopted rather than developed in this study. The primary sources of uncertainty included the stochastic packing density of whole tires and the visual estimation of the burned volume. Additional uncertainty arose from the use of literature-derived emission factors, particularly for products of incomplete combustion generated under oxygen-limited and dynamically suppressed burning conditions. High epistemic uncertainty was assigned to PM, VOC and PAH emissions, as the formation of these pollutants is strongly influenced by transient combustion efficiency, local temperature gradients, and the cooling effects of water and foam application. Consequently, the resulting emission estimates should be interpreted as order-of-magnitude values suitable for source-term characterization and comparative risk analysis rather than as exact quantitative measurements.

### 2.5. Atmospheric Dispersion and Exposure Assessment

The dispersion and potential health impacts of particulate matter (PM10) were evaluated using numerical atmospheric transport models, which provide a robust framework for estimating spatio-temporal pollutant concentrations [8,10]. Several established models were initially considered, including AERMOD, CALPUFF, CAMx, FLEXPART, and HYSPLIT. Previous benchmarking studies across 11 statistical performance metrics have identified CAMx as the most accurate (average rank 1.55), followed by HYSPLIT (average rank 2.27) [11]. However, the implementation of CAMx was precluded by its prohibitive computational demands, requiring dedicated multiprocessor workstations or server-grade infrastructure. Consequently, the HYSPLIT model was selected for its optimal balance between predictive performance and practical applicability. The HYSPLIT simulations were driven by archived GDAS1 meteorological fields (1° × 1°; file gdas1.oct25.w2) for the fire location (52.3883° N, 17.0197° E), using forward trajectories released at 10, 50 and 100 m above ground level to represent the near-surface exposure layer. The emission period was set to 567 min (9 h 27 min), matching the documented duration of active combustion, and a 24 h simulation window was chosen to cover the full transport and deposition phase of the plume (see Text S1). HYSPLIT is integrated with the Real-time Environmental Applications and Display sYstem (READY), enabling the use of high-performance computational resources provided by the National Oceanic and Atmospheric Administration (NOAA) for dispersion simulations [12]. This approach facilitated the efficient processing of archival meteorological data without the need for local high-performance computing (HPC) clusters. HYSPLIT simulates particle trajectories from defined emission sources to determine spatial concentration fields. Dispersion processes were driven by meteorological datasets from NOAA repositories [13], specifically the Global Data Assimilation System (GDAS) with a spatial resolution of 1°. For the purpose of health risk assessment, the model estimated average pollutant concentrations within the near-surface breathing zone (0–100 m above ground level). This vertical layer is consistent with typical human exposure conditions. Furthermore, previous research has demonstrated that vertical concentration gradients remain minimal within this height range [14,15,16]. Nevertheless, this vertical averaging and the assumption of a constant emission rate throughout the simulation period are recognized as inherent limitations of the modeling framework. The strictly technical input parameters and the dense meteorological configurations for the HYSPLIT simulations, derived from the SWD dataset, are detailed in Supplementary Materials to maintain the readability of the methodological narrative.

#### 2.5.1. Plume Rise Parameterisation

For a thermally buoyant source such as a landfill tire fire, HYSPLIT incorporates the Briggs plume-rise algorithm [17], activated via the PLRISE = 1 flag in the model SETUP.CFG. The effective emission height Heff is defined as:

(3) $$H_{eff}=H_{s}+\Delta H$$

where *H_s_* = 1 m is the physical source height (ground-level fire) and Δ*H* is the plume rise computed via the Briggs final-rise equation:

(4) $$\Delta H=1.6\cdot F_{b}^{1/3}\cdot u^{-1}\cdot x_{f}^{2/3}$$

where *u* (m s^−1^) is the mean wind speed at source height and $x_{f}$ (m) is the downwind distance to final plume rise, calculated as:

(5) $$x_{f}=120\cdot F_{b}^{2/5}\cdot u^{-3/5}$$

The buoyancy flux parameter $F_{b}$ (m^4^ s^−3^) was estimated from the fire heat release rate *Q* (W):

(6) $$F_{b}=\frac{\left(g\cdot Q\right)}{\left(\pi \cdot \rho \cdot c_{p}\cdot T_{a}\right)}$$

where *g* = 9.81 m s^−2^, *ρ* = 1.25 kg m^−3^ (ambient air density), $c_{p}$ = 1005 J kg^−1^ K^−1^, and $T_{a}$ = 283 K (ambient temperature retrieved from GDAS1 reanalysis at 02:00 UTC, 13 October 2025). The heat release rate *Q* was derived from the combustion volume (*V* = 450 m^3^), bulk tire density ($\rho_{tire}$ = 100 kg m^−3^), and the higher heating value of vulcanized rubber (*HV* = 32 MJ kg^−1^) integrated over the total fire duration (*t* = 567 min = 34,020 s):

(7) $$Q=\frac{\left(V\cdot \rho_{tire}\cdot HV\right)}{t}=\frac{\left(450\times 100\times 32\times 10^{6}\right)}{34020}\approx 42.3\;MW$$

This yields $F_{b}$ = 371.8 m^4^ s^−3^, a final-rise distance $x_{f}$ = 604 m, and a plume rise Δ*H* = 235 m, resulting in $H_{eff}$= 236 m AGL. These values are consistent with published estimates for large-scale open elastomer fires [5,18] and confirm that the thermal buoyancy was sufficient to inject the fire plume above the nocturnal boundary layer (mixed-layer depth at 02:00 UTC: ~180–220 m, as diagnosed from GDAS1 along the forward trajectories).

The HYSPLIT simulation was initialized with three release heights of 10, 50, and 100 m AGL, consistent with the near-surface human exposure layer defined in Section 2.7. Although these levels lie below Heff, this configuration is deliberate: HYSPLIT’s internal vertical velocity field (KMSL = 0, model vertical velocity) transports particles upward during the active fire phase, while the near-surface levels capture the descending plume during fumigation events. The mean wind speed at source height was estimated at *u* ≈ 3.5 m s^−1^ from GDAS1, and atmospheric stability was assessed as Pasquill–Gifford class D (near-neutral nocturnal conditions). The complete NAMELIST configuration is provided in Supplementary Materials (Text S1).

#### 2.5.2. Vertical Concentration Profiles

To address the vertical structure of the fire plume at increasing downwind distances, PM10 concentration profiles *C*(*z*) were computed using the Gaussian plume dispersion equation [19] for a ground-reflected elevated source [5]:

(8) $$C\left(x,z\right)=\frac{Q_{PM}}{\pi \cdot u\;\cdot \sigma_{y}\cdot \sigma_{z}}\cdot \exp\left[-\frac{1}{2}{\left(\frac{z-H_{eff}}{\sigma_{z}}\right)}^{2}\right]+\exp\left[-\frac{1}{2}{\left(z+\overset{\sigma_{z}}{H_{eff}}\right)}^{2}\right]$$

where *C*(*x*, *z*) is the pollutant concentration at downwind distance *x* and height *z*, *σ_y_* and *σ_z_* (m) are the lateral and vertical dispersion coefficients calculated using the Pasquill–Gifford Briggs rural parameterisation for stability class D: *σ_y_* = 0.08 × (1 + 0.0001*x*) − 0.5 and *σ_z_* = 0.06 × (1 + 0.0015*x*) − 0.5, with *x* in meters. The PM10 mass emission rate *Q_PM_* = 39.4 g s^−1^ was derived from the total PM10 emission (1340 kg) integrated over the fire duration (34,020 s).

**Figure 2:**
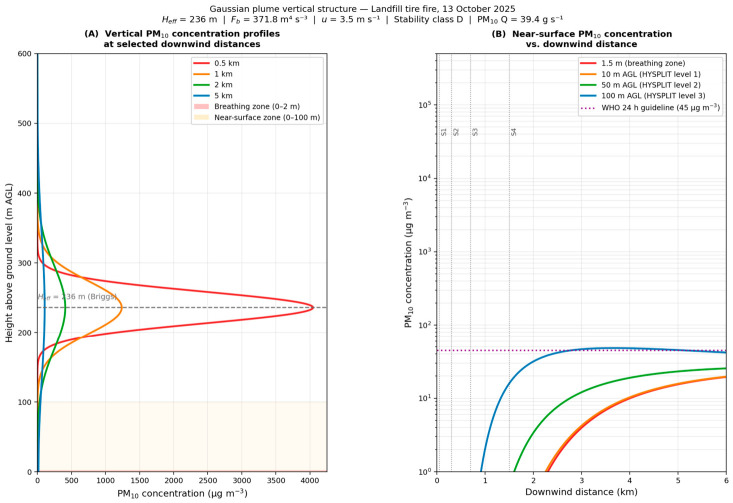
(**A**) Vertical PM10 concentration profiles at selected downwind distances, computed using the Gaussian plume model (Equation (8)) with Briggs plume-rise parameters derived from the fire event (Heff = 236 m, QPM = 39.4 g s^−1^, stability class D). The breathing zone (0–2 m AGL) and the near-surface HYSPLIT monitoring layer (0–100 m AGL) are shaded. (**B**) Near-surface PM10 concentrations as a function of downwind distance at the three HYSPLIT trajectory release heights (10, 50, 100 m AGL) and at breathing-zone level (1.5 m AGL). The WHO 24 h PM10 guideline (45 µg m^−3^) and approximate locations of soil sampling sites S1–S4 are indicated.

The resulting profiles at downwind distances of 0.5, 1, 2, and 5 km are presented in Figure 2. Three key observations emerge: (i) within 1 km of the source, the concentration maximum remains near Heff (~236 m AGL), effectively decoupling the elevated plume from the breathing zone and explaining the low near-surface concentrations at the fire scene during active combustion; (ii) beyond 2 km, progressive turbulent diffusion transports the plume downward, and near-surface PM10 concentrations approach or exceed the WHO 24 h guideline of 45 µg m^−3^, consistent with the concentration spikes recorded at the regional monitoring stations (Figure 2); (iii) the relative concentration difference between the three HYSPLIT monitoring levels (10, 50, and 100 m AGL) does not exceed 15% at distances beyond 1 km from the source (Figure 2B), thereby validating the vertical layer-averaging approach adopted for human exposure assessment in Section 2.7.

### 2.6. Chemical Analysis

#### 2.6.1. PAHs

Analysis of polycyclic aromatic hydrocarbons (PAHs) in soil and surface water was commissioned to an accredited laboratory (ISO/IEC 17025 [20]). PAHs were extracted from soil samples using supercritical fluid extraction (SFE) with CO_2_ modified by acetone (25 MPa, 50 °C, 30 min), followed by concentration under nitrogen stream. Water samples underwent solid-phase extraction (SPE) on C18 cartridges preconditioned with methanol and Milli-Q water, eluted with dichloromethane:acetone (9:1, *v*/*v*). Extracts were exchanged to hexane, fractionated on silica gel columns (hexane:dichloromethane 7:3), and concentrated to 100 µL. The 16 priority PAHs (e.g., naphthalene, benzo[a]pyrene) were quantified via gas chromatography–mass spectrometry (GC-MS) in selected ion monitoring (SIM) mode using a DB-5MS column (30 m × 0.25 mm × 0.25 µm), temperature program 60 °C (1 min) to 300 °C at 8 °C/min, with deuterated surrogates (e.g., D8-naphthalene) for quality control. Method detection limits were 0.1–0.5 µg/kg (soil) and 5–20 ng/L (water), with repeatability (RSD < 10%) and recoveries of 85–105% confirmed by fortified blanks and reference materials.

#### 2.6.2. Trace Metals

The elements were determined by inductively coupled plasma–mass spectrometry (ICP-MS) on the Agilent Technologies 7700x (Agilent Technologies, Santa Clara, CA, USA) series apparatus as described previously [21,22]. The multi-element certificated reference materials for Rare Earth Element Mix from TraceCERT 16 elements, 50 mg/L in nitric acid (Sigma-Aldrich No. 67349, St. Louis, MO, USA), and trace elements from Certipur^®^ was used for calibration. The accuracy of the method expressed as the level of analyte recovery ranged from 91.1% to 98.6%. The instrumental limit of quantification (LOQ) was set at 1 ppb.

### 2.7. Human Health Risk Assessment

In this study, TEQ and MEQ are utilized to characterize the intrinsic toxicological and mutagenic hazard of the contaminated soil matrix itself (a source-centric assessment). In contrast, the ILCR framework is employed to quantify the receptor-specific probability of cancer development (a receptor-centric assessment). Both are necessary: TEQ/MEQ provide a standardized metric of environmental degradation independent of human behavior, whereas ILCR accounts for physiological parameters, exposure durations, and multi-pathway uptake (ingestion, dermal, inhalation) critical for human health risk management [19,21,23].

#### 2.7.1. Exposure Scenarios and Pathways

The population was categorized into two groups: children (1–6 years) and adults (7–70 years), reflecting physiological and behavioral differences in exposure. Three primary pathways were integrated to estimate the cumulative risk: direct Ingestion (accidental intake of contaminated soil/dust particles), dermal contact (absorption of PAHs through the skin surface) and inhalation (intake of resuspended particles and volatile fractions within the fire-impacted zone).

#### 2.7.2. Quantitative Risk Model

The cumulative Incremental Lifetime Cancer Risk (∑ILCR) was calculated by determining the risk for each individual PAH (i) across each specific exposure pathway, and then aggregating the results to avoid double-counting. We deliberately applied compound-specific Cancer Slope Factors (CSFs) directly to the concentration of each PAH rather than using Toxic Equivalency Factors (TEFs) in the ILCR equations, maintaining maximum resolution of individual risk drivers. The standard deterministic equations for exposure assessment were adopted from U.S. EPA guidelines [24] and implemented following the methodology described by Yuan et al. [25] and Deelaman et al. [26]:

(9) $$ILCR_{ing}=\frac{C_{s}\times IngR\times EF\times ED}{BW\times AT}\times CSF_{ing}$$

(10) $$ILCR_{derm}=\frac{C_{s}\times SA\times AF\times ABS\times EF\times ED}{BW\times AT}\times \left(\frac{CSF_{ing}}{GIABS}\right)$$

(11) $$ILCR_{inh}=\frac{C_{s}\times InhR\times EF\times ED}{PEF\times BW\times AT}\times CSF_{inh}$$

The cumulative risk was defined as

(12) $$\sum ILCR=\sum_{i=1}^{n}\left(ILCR_{ing},i+ILCR_{derm},i+ILCR_{inh},i\right)$$

where *C_s_* is the concentration of the specific PAH in soil (mg/kg); *IngR* is the ingestion rate (mg/day); *InhR* is the inhalation rate (m^3^/day); *SA* is the exposed skin surface area (cm^2^); *AF* is the soil adherence factor (mg/cm^2^); *ABS* is the dermal absorption fraction (unitless); *EF* is the exposure frequency (days/year); *ED* is the exposure duration (years); *BW* is the body weight (kg); *AT* is the averaging time (days); *PEF* is the particulate emission factor (m^3^/kg); *GIABS* is the gastrointestinal absorption factor (unitless); and *CSF_ing_* and *CSF_inh_* are the oral and inhalation cancer slope factors (mg/kg·day)^−1^, respectively.

### 2.8. Ecological Risk Assessment

Terrestrial ecological risk was quantified using the Risk Quotient (RQ) framework, comparing measured PAH concentrations against toxicological benchmarks [23,26,27]. Maximum Permissible Concentrations (MPCs) were adopted from national soil quality standards [28], ensuring site-specific regulatory relevance.

To account for chronic exposure and latent mixture effects, a conservative tier was integrated by defining Negligible Concentrations (NCs) as 1/100 of the MPC [27]. Given the co-occurrence of multiple pyrogenic pollutants, cumulative ecological risk was evaluated through a concentration addition model [29,30], allowing for the identification of both individual chemical drivers and the aggregate environmental burden posed by the fire-impacted PAH suite.

### 2.9. Statistical and Multivariate Analysis

In order to further interpret the results obtained and to identify the relationship between the concentrations of elements and polycyclic aromatic hydrocarbons (PAHs) and location, the chemical data were subjected to advanced multivariate analysis (PCA). All calculations and visualizations were performed using PAST (Paleontological Statistics, version 4.16) software.

Due to the specificity of the on-site research, the analysis included data from 5 key locations (*N* = 5). Due to the fact that the analytes examined represented extremely different orders of magnitude (from macroelements naturally occurring in the soil to trace organic pollutants), the data were subjected to automatic autoscaling (Z-score standardization) before proceeding with the actual analyses. This procedure equalized the mathematical weight of all variables, making it possible to study the actual correlations rather than the numerical dominance of individual elements. Principal Component Analysis (PCA) was performed, based on the correlation matrix. Due to the specific structure of the data set (a large number of chemical parameters to be determined with a limited number of measurement points), the correlation matrix was characterized by a zero determinant, which made it impossible to determine traditional measures of sample adequacy, such as the Bartlett sphericity test or the KMO index.

To evaluate the variance structure, a “broken stick model” test was used. However, it is crucial to acknowledge that given the very limited number of spatial samples (*N* = 5), the PCA is utilized in this study strictly as an exploratory tool to visualize broad chemical gradients and potential source grouping, rather than to provide robust statistical evidence. This criterion showed that the first two principal components (PC1 and PC2) fully exceed the theoretical values generated by random noise, explaining a total of as much as 93% of the total variance in the data. This allowed for a safe reduction in dimensionality to a two-dimensional factor space (PC1 vs. PC2) and a spatial interpretation of the gradient of contaminants in the studied soil profiles.

## 3. Results

### 3.1. Quantification of Source Emissions and Uncertainty Profile

The estimated pollutant mass loadings (Table 1) reveal a substantial atmospheric burden generated during the fire event. Carbon dioxide (CO_2_) was the predominant species, with a total release of 45.5 ± 26.4 t, serving as a proxy for the total carbon-rich polymer mass consumed. However, the high emissions of carbon monoxide (1.12 t) and PM_10_ (1.34 t) underscore a highly inefficient, oxygen-limited combustion regime, typical of compacted waste tire piles. The SO_2_ flux (441 kg) is consistent with the oxidative release of sulphur cross-linking agents used in the tire vulcanization process. The uncertainty analysis (Table 1) indicates a significant gradient in confidence levels across the pollutant suite. While CO_2_ showed the lowest relative uncertainty (57.9%), products of incomplete combustion (PICs), specifically PM_10_ (93.5%), VOCs (114.6%), and ∑ PAHs (160.1%), exhibited markedly higher variance. This reflects the stochastic nature of “cold” smoldering phases and the heterogeneous suppression effects of firefighting foam, which drastically influence the emission factors of organic aerosols and gaseous intermediates. Combining operational SWD data with the emission model indicates that this single fire released 45.5 ± 26.4 t of CO_2_, 1.12 t of CO, 1.34 t of PM_10_ and 17.7 kg of ∑ PAHs, including 0.606 kg of benzo[a]pyrene, over less than 10 h. These values, derived from a combustion volume of approximately 450 m^3^ and a heat release rate of 42.3 MW (see Text S1), underline the catastrophic nature of the event from both an air quality and soil contamination perspective.

### 3.2. Speciation and Distribution of PAHs

Speciated PAH emissions (Table 2) were dominated by low-molecular-weight (LMW) compounds, with Naphthalene (7.34 kg), Phenanthrene (3.53 kg), and Acenaphthylene (3.10 kg) accounting for over 75% of the total PAH mass. These findings align with the thermal degradation profiles of synthetic rubber, where fragmented aromatic precursors are abundant in the gas phase.

The uniform relative uncertainty of 160.1% observed for all individual PAHs and CH_4_ is a mathematical artifact of the error propagation model (Equation (2)). Because the variance in the estimated fuel volume and bulk density is constant for the entire event, and a uniformly high emission factor uncertainty (uEF) was conservatively applied to all trace products of incomplete combustion (PICs) due to the unpredictable quenching effects of firefighting foam, the resulting relative uncertainty yields an identical percentage across these specific species.

Of particular toxicological concern are the high-molecular-weight (HMW) species, including Benzo(a)pyrene (0.606 kg) and Benz(a)anthracene (0.547 kg). Despite their lower mass contribution compared to LMW PAHs, their substantial emission fluxes (>0.5 kg each) pose a disproportionate risk to air quality and human health due to their high toxic potency. The consistency in relative uncertainty (160.1%) across all PAH species (Table 2) originates from the high variability in the adopted emission factors, which are sensitive to the transient combustion temperatures and the cooling effect of the water–foam interface.

### 3.3. Atmospheric Transport and Ground-Level Air Quality Response

**Figure 3:**
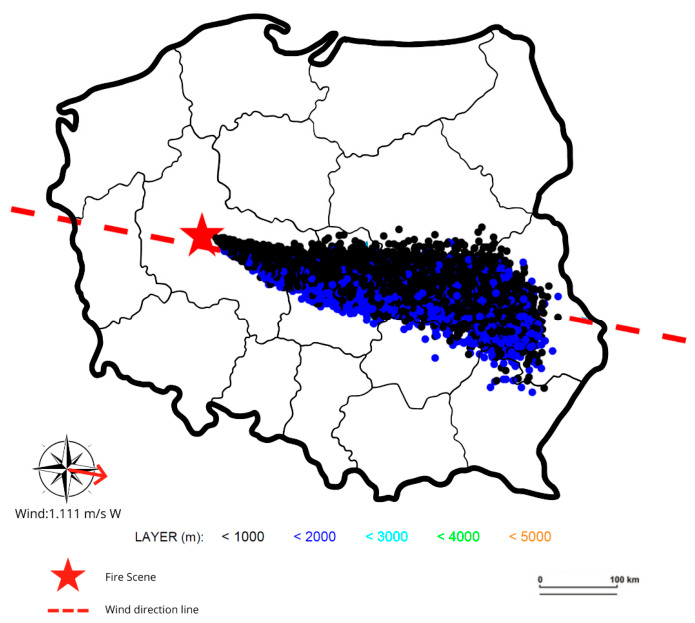
HYSPLIT forward trajectories and plume dispersion. Simulation of the fire plume transport pathways during the initial 24 h. The trajectories represent the primary downwind sector and the spatial extent of fire-related pollutant dispersion.

Atmospheric dispersion simulations performed with the HYSPLIT model revealed a coherent transport pattern for the fire-generated plume, controlled primarily by the prevailing synoptic conditions (Figure 3). Forward trajectory analysis identified a dominant downwind sector where plume transport within the planetary boundary layer indicated an increased likelihood of near-surface impacts. These spatial modeling results provided a defensible basis for evaluating potential population exposure and for selecting representative air quality monitoring locations.

**Figure 4:**
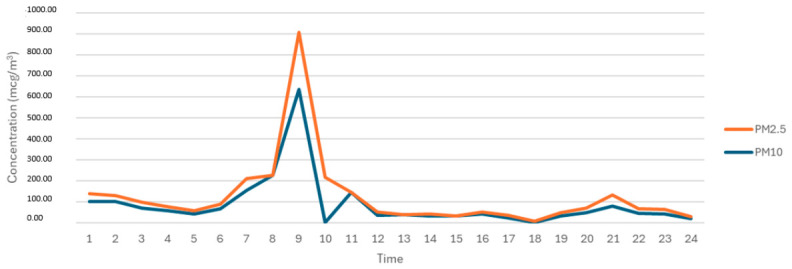
Hourly PM_2.5_ and PM_10_ concentration profiles before, during, and after the fire event. Shaded regions denote the simulated plume passage, illustrating the temporal correlation between emissions and air quality spikes.

Based on the simulated plume footprint and anemometric characteristics, three monitoring stations situated along the primary transport axis were analyzed. Temporal profiles of PM_2.5_ and PM_10_ across these sites showed distinct concentration spikes that synchronized with the simulated plume passage (Figure 4). At all selected nodes, a marked departure from pre-fire background levels was observed, confirming the influence of the tire landfill fire on regional air quality.

The magnitude and duration of these pollution episodes exhibited a clear spatial gradient. Peak concentrations were most pronounced at stations proximal to the plume centreline, where the dilution effect was minimized. Conversely, stations located at greater downwind distances or lateral displacements showed more attenuated and temporally shifted responses, reflecting the combined effects of dry deposition and atmospheric dispersion. Collectively, these integrated modeling and observational results demonstrate a spatially heterogeneous but temporally coherent impact of the fire event on the local atmospheric environment.

### 3.4. Elemental and PAH Composition in Soil Matrices

The elemental analysis (Table 3) demonstrated a sharp spatial attenuation of contaminants from the fire origin (S1) towards the reference site (S5). Location S5, situated upwind/crosswind, exhibited concentrations consistent with regional geochemical background levels, providing a baseline for assessing the impact of the fire event. Zinc (Zn) and Copper (Cu) showed extreme enrichment at the fire scene (S1), with Zn reaching 26-fold increase relative to the background concentration at S5. This extreme loading is a direct chemical signature of tire rubber degradation. Interestingly, the immediate downwind site (S2) showed peak concentrations for Lead (Pb), Barium (Ba), and Tin (Sn), exceeding the levels found at the ignition point (S1). This suggests that these elements were efficiently mobilized within the thermal plume and deposited shortly after emission. Even at the distal allotment gardens (S4), Zn levels (349.6 mg/kg dw) remained significantly higher than the background (S5), indicating that fire-related particulates reached residential areas. The ∑ PAH profile confirmed a similar trend. The total concentration at the fire site (S1: 148.9 mg/kg dw.) was two orders of magnitude higher than the reference site (S5: 2.69 mg/kg sm). While S5 showed a typical urban/suburban background signature, the elevated PAH levels at S4 demonstrate substantial pyrogenic deposition in areas used for food production.

### 3.5. Multivariate Analysis of Source Apportionment (PCA)

Analysis of factor charge values showed that the first principal component (PC1) represents the total level of chemical anthropopressure in the soil. All analytes examined obtained positive load values on this axis. The highest contribution to the structure of PC1 was made by polycyclic aromatic hydrocarbons with a higher molecular weight, including dibenzo(a,h)anthracene (0.27), indeno(1,2,3-cd)pyrene (0.26), benzo(b)fluoranthene (0.24) and chrysene (0.23), as well as trace metals, namely zinc (0.26), copper (0.25) and cadmium (0.24).

In turn, the second main component (PC2) revealed a clear polarization and distinctiveness of the sources of origin of organic and inorganic pollutants. Positive load values on the PC2 axis were dominated by elements and metalloids of a potential industrial nature or strongly associated with the soil matrix, such as iron (0.31), arsenic (0.30), chromium (0.30), barium (0.30) and nickel (0.28). On the opposite, negative side of the PC2 axis, only compounds from the PAH group were grouped, in particular benzo(k)fluoranthene (−0.19), anthracene (−0.18), benzo(a)pyrene (−0.18) and chrysene (−0.17).

**Figure 5:**
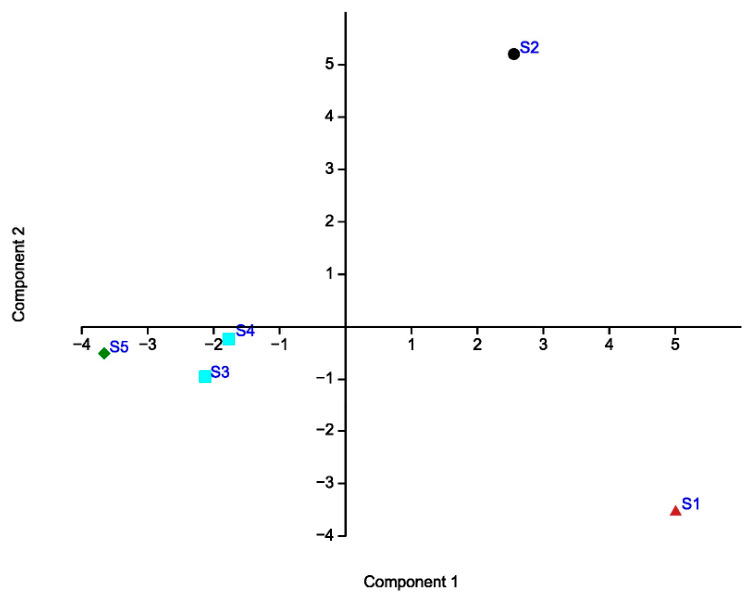
Principal Component Analysis (PCA) biplot of soil samples (S1–S5) based on elemental concentrations and polycyclic aromatic hydrocarbons (PAHs) profiles. Values in parentheses indicate the percentage of total variance explained by PC1 and PC2.

Projection of the coordinates of the measuring stations onto the PC1 vs. PC2 plane allowed for the unambiguous separation of three separate environmental zones (Figure 5). At Position S1 (PC1 = 5.01; PC2 = −3.51), the site was characterized by the highest overall degree of organic pollution, dominated by the emission of PAHs with a pyrolytic profile. Position S2 (PC1 = 2.55; PC2 = 5.20) showed an extremely different chemical profile, determined by the extraordinary accumulation of heavy metals and metalloids, with a relatively lower share of hydrocarbons. Sites S3, S4 and S5 formed a compact group in the negative quadrant of the PC1 axis, which classifies them as areas of low anthropopressure. Among them, the S3 site (PC1 = −2.13) was closest to the center of the system, suggesting the initial impact of the migration of pollutants from the area covered by the event. Point S5 (PC1 = −3.66) showed the most extreme negative coordinates, confirming its role as a representative, pure reference background for the soils studied.

### 3.6. Toxicological and Mutagenic Potency (TEQ and MEQ)

The ∑TEQ values were unequivocally dominated by Benzo(a)pyrene (BaP), which acted as the primary driver of carcinogenic potency across all samples. At the fire origin (S1), BaP accounted for 73.1% relative contribution (18.67 mg BaPeq/kg) of the total TEQ (25.52 mg BaPeq/kg). Other significant contributors included Dibenzo(a,h)anthracene (7.2%) and Benzo(a)anthracene (6.0%). This profile remained consistent even at the distal allotment gardens (S4), where BaP still contributed over 67% to the local TEQ, confirming that the high-molecular-weight (HMW) PAHs are the critical determinants of long-term health risks associated with tire fire residues.

**Figure 6:**
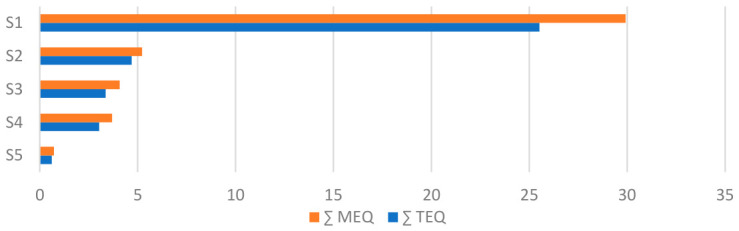
Spatial distribution and absolute values of mutagenic equivalents (∑MEQ) and toxic equivalency (∑TEQ) across the sampling sites.

The ∑MEQ followed a similar spatial distribution but yielded higher absolute values than TEQ (Figure 6), peaking at 29.92 mg/kg at S1. The mutagenic profile was similarly dominated by Benzo(a)pyrene, but with a more pronounced influence from Benzo(b)fluoranthene. At the fire site, Benzo(b)fluoranthene contributed 22.3% to the total MEQ, significantly more than its contribution to the TEQ (10.5%). Other compounds with notable mutagenic impact were Benzo(k)fluoranthene and Indeno(1,2,3-cd)pyrene.

Those findings illustrate a serious “toxicological cliff” between the fire zone (S1) and the reference background (S5). While S5 represents a low-level urban baseline (∑TEQ = 0.62; ∑MEQ = 0.72), the values at the allotment gardens (S4) remain nearly five times higher than the background. This elevation is primarily driven by the deposition of PAHs, which, due to their low volatility and high persistence, accumulate in the upper soil layer, posing a sustained risk to the local community.

### 3.7. Human Health Risk Assessment

The incremental lifetime cancer risk (ILCR) calculated for the tire fire event exhibited a pronounced spatial gradient and pathway-specific variability, as summarized in Table 4. Benchmark analysis against the U.S. EPA safety standards revealed that the cumulative risk (∑ILCR) at the fire scene (S1) reached 8.3 × 10^−4^ for adults and 9.7 × 10^−4^ for children, significantly exceeding the 10^−4^ threshold for significant health intervention. This elevated risk profile was primarily driven by the dermal pathway, which emerged as the dominant exposure route at all fire-impacted sites (S1–S4). At the point of ignition (S1), dermal ILCR values for individual compounds such as Naphthalene (1.59 × 10^−4^) and Benzo(b)fluoranthene (1.59 × 10^−4^) for children surpassed the critical safety limit, while ingestion risks remained an order of magnitude lower, albeit still within the 10^−6^ to 10^−5^ low-risk range.

As the distance from the source increased, the carcinogenic risk showed a rapid but uneven attenuation. At the immediate downwind location (S2), the cumulative risk remained above the 10^−4^ threshold (1.4 × 10^−4^ to 1.6 × 10^−4^), maintained largely by the dermal absorption of high-molecular-weight PAHs deposited from the smoke plume. Further downwind at the allotment gardens (S4), the total ILCR values ranged between 8.0 × 10^−5^ and 1.0 × 10^−4^. These values fall within the 10^−6^ to 10^−4^ interval, characterizing the residential and gardening zone as a moderate-risk area. In contrast, the ILCR at the background station (S5) was consistently the lowest, with all pathways yielding values near or below the 10^−6^ negligible risk level, thereby isolating the fire event as the primary source of the observed toxicological burden.

A comparative analysis between population groups revealed that children are subject to higher integrated risks than adults across the ingestion and dermal pathways. At the fire scene (S1), the ingestion-related ILCR for children was 1.6 times higher than that recorded for adults. However, a reversal of this trend was observed for the inhalation pathway, where adults exhibited higher risk values (e.g., 5.91 × 10^−6^ vs. 2.47 × 10^−6^ for Naphthalene at S1), reflecting the higher physiological breathing rates and exposure durations assumed for the adult demographic. These results collectively demonstrate that while the most severe health risks are localized near the fire origin, the deposition of pyrogenic PAHs has elevated the carcinogenic risk in distal residential areas to levels that necessitate long-term monitoring.

### 3.8. Ecological Risk Assessment

The ecological impact of the fire event was quantified through Risk Quotients (RQ) based on both Maximum Permissible Concentrations (MPC) and Negligible Concentrations (NCs), as detailed in Table 5. The analysis of trace elements revealed that Zinc (Zn) and Copper (Cu) posed the most significant environmental stress. At the fire scene (S1) and the immediate downwind site (S2), RQ_(MPC)_ values for Zn reached 1.50, corresponding to a critical RQ_(NCs)_ of 150, which indicates an extreme departure from baseline ecological safety levels. Similarly, Cu exhibited an RQ_(MPC)_ > 1 at S2 (1.03), suggesting that the fire-generated plume and runoff significantly compromised the soil quality in the immediate vicinity. Other elements, including Cobalt (Co) at S1 (RQ_(MPC)_ = 0.69) and Lead (Pb) at S2 (RQ_(MPC)_ = 0.59), although below the unity threshold for MPC, showed high RQ_(NCs)_ values (68.50 and 58.67, respectively), highlighting potential long-term chronic risks to soil biota.

The ecological risk posed by PAH mixtures was markedly higher than that of individual trace elements. The cumulative risk for the PAH suite (∑RQ_(MPC)_) remained above the safety threshold at all sampling points, ranging from 1.06 at S3 to a peak of 6.91 at the fire origin (S1). Benzo(a)pyrene (BaP) was identified as the primary ecological risk driver, with an individual RQ_(MPC)_ of 3.06 at S1, translating to an RQ_(NCs)_ of 306.25. Notably, even at the distal allotment gardens (S4), the RQ_(MPC)_ for BaP (1.65) exceeded the permissible limit, indicating that the deposition of high-molecular-weight PAHs from the thermal plume has created an ecologically unstable environment in residential gardening areas.

Comparison with the background site (S5) underscores the severity of the pyrogenic impact. While S5 displayed high individual RQ values for certain PAHs, reflecting a typical urban baseline for compounds like BaP (RQ_(MPC)_ = 8.00), the cumulative RQ_∑PAHs_ at the fire-impacted sites (S1–S4) consistently represented a significant additional toxicological burden. The spatial distribution of risk confirms that while elements like Zn and Cu provide a localized chemical signature of tire combustion, the PAH suite, particularly BaP and Benzo(b)fluoranthene, extends the ecological risk to a broader spatial scale, necessitating a conservative approach to long-term soil management in the affected downwind sector.

## 4. Discussion

Tire combustion generates a complex mixture of hazardous gases and particulate pollutants, the composition of which depends on conditions such as oxidant availability and temperature. By weight, tires typically consist of approximately 50% natural or synthetic rubber, 25% carbon black, around 10% metals (primarily associated with steel reinforcement), and smaller fractions of sulphur, zinc oxide, and various additives [31].

Consequently, combustion of this heterogeneous material results in the release of a broad spectrum of pollutants. Laboratory combustion studies have reported substantial emission factors for CO_2_ (2890 g kg^−1^), CO (71 g kg^−1^), NO_x_ (6.0 g kg^−1^), and SO_2_ (28 g kg^−1^) [5], alongside significant emissions of total suspended particulates (TSP, 65–105 g kg^−1^), gas- and particle-phase PAHs (3.4–5.3 g kg^−1^), and volatile organic compounds (12–50 g kg^−1^), including benzene, toluene, and xylene [9].

The environmental investigation of large-scale scrap tire fires necessitates an advanced understanding of the thermal degradation of vulcanized elastomers, primarily styrene–butadiene rubber (SBR). Unlike controlled industrial incineration, tire stockpile fires typically occur under oxygen-limited conditions. Despite the open-air setting, the high thermal mass of the fuel creates an internal environment characterized by elevated temperatures and low bulk air ratios. Under these fuel-rich conditions, the thermolysis of SBR promotes the de novo synthesis of a vast and complex spectrum of aromatic hydrocarbons rather than complete oxidation to carbon dioxide [4]. Laboratory studies using horizontal reactors have confirmed this mechanism, demonstrating that decreasing the air ratio significantly increases the yield of volatile and semi-volatile organic compounds [32]. Field investigations following large-scale events have confirmed the resulting complexity of these combustion residues, which far exceeds the scope of standard environmental monitoring. While common protocols focus on the 16 priority polycyclic aromatic hydrocarbons (PAHs), forensic characterization of significant events, such as the Gatineau tire fire involving approximately 6000 new and recycled tires, has identified upwards of 165 PAH-related compounds in soot and liquid residues [33]. This mixture is characterized by a high abundance of high-molecular-weight (HMW) isomers. These HMW structures, many of which contain sulphur-, nitrogen-, and oxygen-substituted groups, serve as unique pyrogenic fingerprints for tire-derived soot [33]. Given that many of the compounds emitted during tire combustion are toxic, carcinogenic, or mutagenic, these events represent a significant environmental and public health concern. Indeed, mutagenicity assays have demonstrated that tire combustion emissions may exhibit higher mutagenic potential than emissions from open plastic burning of fossil fuel combustion in utility boilers [34]. In the context of the present study, these chemical and thermodynamic characteristics help explain the elevated concentrations of PAHs observed in affected soils and post-fire runoff, as well as the associated toxic equivalency (TEQ) and human health risk estimates.

The uncontrolled combustion of scrap tires in landfill presents a singular challenge for atmospheric chemists and environmental risk assessors due to the complex interplay between polymer degradation, heterogeneous oxidation, and the chaotic influence of firefighting interventions. The recent quantification of source emissions from such an event reveals a substantial atmospheric burden, where CO_2_ release acts as a primary indicator of total polymer consumption, while a suite of products of incomplete combustion (PICs) defines the toxicological and environmental hazard. The data indicates that during this specific fire event, approximately 45.5 ± 26.4 t of carbon dioxide (CO_2_) was emitted, serving as a critical proxy for the total mass of carbon-rich tire material consumed. However, the concurrent emission of 1.12 t of carbon monoxide (CO) and 1.34 t of PM_10_ highlights a highly inefficient and oxygen-limited combustion regime, which is the hallmark of compacted waste tire piles [5].

Polycyclic Aromatic Hydrocarbons (PAHs) represent the most toxic fraction of tire fire emissions. They are formed through pyrosynthesis, the recombination of small hydrocarbon fragments into fused ring structures, under oxygen-starved, high-temperature conditions. The reported PAH emissions are dominated by low-molecular-weight (LMW) compounds, with Naphthalene (7.34 kg), Phenanthrene (3.53 kg), and Acenaphthylene (3.10 kg) accounting for over 75% of the total ∑ PAH mass.

The abundance of LMW PAHs (2–3 rings) is characteristic of the thermal degradation of synthetic rubber. In a tire fire, the breakdown of styrene–butadiene rubber (SBR) releases benzyl and phenyl radicals, which are key precursors for the formation of naphthalene and larger aromatics. Because LMW PAHs have higher vapor pressures, they are predominantly found in the gas phase or as weakly associated vapor-phase compounds in the plume. Naphthalene, in particular, is a ubiquitous product of incomplete combustion and often serves as a marker for the overall intensity of the organic flux.

While LMW PAHs dominate the mass loading, the high-molecular-weight (HMW) species (4–6 rings) carry the highest toxicological potency. Benzo(a)pyrene (BaP) and Benz(a)anthracene (BaA), with emission fluxes exceeding 0.5 kg each, are of primary concern due to their carcinogenic, mutagenic, and teratogenic properties. BaP is frequently used as the “gold standard” index for determining the cumulative toxicity of a PAH mixture through the Relative Potency Factor (RPF) or Toxic Equivalency Factor (TEF) approach [35,36].

The relative uncertainty of 160.1% associated with individual PAH emissions reflects the high variability inherent to uncontrolled landfill fires. This uncertainty is primarily driven by fluctuations in combustion conditions and emission factors rather than analytical limitations. Therefore, the calculated PAH emissions should be interpreted as order-of-magnitude estimates. Despite the wide uncertainty range, the presence and estimated release of carcinogenic high-molecular-weight PAHs such as benzo[a]pyrene and benzo[b]fluoranthene confirm that landfill tire fires represent a significant episodic source of health-relevant pollutants.

While LMW PAHs dominated the mass profile, the identification of HMW isomers suggests a complex pyrogenic fingerprint. The presence of 13 isomers of MW 302 and 10 isomers of MW 278 is a stable forensic marker of scrap tire thermolysis [33]. Standard monitoring restricted to 16 compounds significantly underestimates the total aromatic burden and chronic risk associated with tire fire fallout.

The synchronization of PM concentration spikes with HYSPLIT modeling validates the “source-to-receptor” model. Large fires generate sufficient buoyancy to penetrate the capping inversion of the planetary boundary layer (PBL), injecting contaminants into the free atmosphere. The observed spatial gradient confirms the “fumigation” effect, where pollutants transported aloft are mixed down to the surface, creating distal hotspots far from the source [37].

The 26-fold increase in Zinc (Zn) is a direct chemical fingerprint of the ZnO vulcanization activator [38,39]. To definitively separate fire fallout from chronic traffic abrasion, forensic science relies on the correlation between Zn and Selenium (Se). While Zn is common in roadside soil due to mechanical tire wear, the thermal vaporization and subsequent adsorption of Se onto fine particulates create a unique co-marker profile specific to pyrogenic events [18,25].

This pyrogenic signature is further supported by our exploratory our Principal Component Analysis (PCA). While interpreted with caution due to the limited sample size, the strong positive loading of both high-molecular-weight PAHs (e.g., dibenzo(a,h)anthracene, chrysene) and specific inorganic tracers (Zn, Cu) along the PC1 axis suggests that the total chemical anthropopressure at the fire scene (S1) and the immediate downwind runoff pathways (S2) share a unified, event-driven origin. Furthermore, the spatial polarization revealed by the exploratory PCA visually separates the S1 site as the pyrolytic core, S2 as the heavy metal sink (driven by firefighting runoff), and establishes the distal sampling points (S3–S4) along a structured attenuation gradient towards the unimpacted background (S5). This exploratory multivariate analysis further supports the premise that the elevated health risks calculated for the allotment gardens are tied to the tire fire deposition footprint rather than historical, unrelated urban contamination.

The higher absolute values of MEQ compared to TEQ reveal a hidden mutagenic hazard. This shift is driven by compounds like Benzo(b)fluoranthene and Indeno [1,2,3-cd]pyrene, which have higher Mutagenic Equivalency Factors (MEFs of 0.25 and 0.31) than their Toxic Equivalency Factors (TEFs) [40]. This disparity is critical for assessing the DNA damage potential in populations experiencing acute episodic exposure [6].

While inhalation is the most immediate threat, dermal exposure is a significant concern for first responders and clean-up crews. PAHs like naphthalene are readily absorbed through the skin, and the extreme conditions of firefighting, including heat and sweat, can accelerate this process. Furthermore, the particulate matter (PM_10_) emitted is linked to increased emergency hospital admissions for lung and heart disease, with every 10 μg/m^3^ increase in 24 h concentration correlating with a rise in daily mortality [41].

The calculated Incremental Lifetime Cancer Risk (ILCR) and Risk Quotient (RQ) for this fire event reflect the high toxicological burden generated by elastomer combustion. Total emissions of 16 priority polycyclic aromatic hydrocarbons (PAHs) reached 17.7 kg, with substantial contributions from high-molecular-weight (HMW) species such as Benzo(a)pyrene (0.606 kg) and Benz(a)anthracene (0.547 kg).

The ILCR values, often exceeding the generally acceptable threshold of 10^−6^, are characteristic of uncontrolled landfill fires. This is underscored by the high flux of Benzo(a)pyrene (BaP), which serves as the “gold standard” index for determining the cumulative toxicity of PAH mixtures [42,43]. In comparable events, such as the 2016 Seseña tire fire in Spain, cancer risks for the population living within 500 m of the landfill were 3 to 5 times higher than for inhabitants in background areas [44].

To contextualize these findings within international environmental regulatory frameworks, the observed contamination levels far exceed standard screening benchmarks. The European Union currently lacks a unified, legally binding directive for numeric PAH limits in soil, often deferring to member state legislation or established international benchmarks [24]. However, comparing our data to widely accepted global standards—such as the U.S. EPA Regional Screening Levels (RSLs) for residential soils [24] or the Dutch Soil Quality Intervention Values (often utilized as a generalized European benchmark for severe contamination)—reveals a critical exceedance. For instance, the U.S. EPA RSL for Benzo(a)pyrene in residential soil is established at a highly conservative 0.11 mg/kg.

At our distal allotment garden site (S4), the BaP concentration was 2.05 mg/kg, nearly 20 times the screening level. This demonstrates that applying standard environmental regulatory thresholds to episodic tire fire events identifies these areas as requiring immediate post-incident land-use management and potential remediation to protect public health.

The IARC recently reclassified occupational exposure as a firefighter as ‘carcinogenic to humans’ (Group 1), identifying PAHs as a primary causal hazard [45]. Our site-specific ILCR calculations translate this recognized hazard into quantifiable risk, demonstrating that the probability of carcinogenic outcomes remains unacceptably high even in distal, non-occupational exposure scenarios (e.g., residential allotment gardens) due to the environmental persistence of these pyrogenic compounds. While inhalation is conventionally the most monitored pathway, recent biomonitoring and toxicological reviews emphasize that dermal contact is a critical and often dominant route of internal PAH dosing for first responders. During and after firefighting activities, the skin is continuously exposed to PAH contamination, primarily on less protected areas such as the neck, wrists, face, and hands [46].

Once deposited, these compounds pose an ongoing threat. Studies utilizing semipermeable membranes and tape-stripping techniques on firefighters have confirmed that both gaseous and particle-bound PAHs, including alkylated species, strongly adhere to the skin surface (e.g., wrists and collarbones) and are not easily removed [45]. Furthermore, while high-molecular-weight (HMW) PAHs dominate the overall carcinogenic risk profile, low-molecular-weight (LMW) species such as Naphthalene (7.34 kg) present exceptionally efficient dermal exposure pathways. Naphthalene has been shown to exhibit a high dose absorption efficiency of 35.0 ± 4.6 when in contact with sweaty skin [41].

This high permeability is fundamentally linked to the lipophilic nature of lighter PAHs, which demonstrate significantly increased dermal permeabilities and absorption rates compared to heavier compounds. Once they penetrate the skin tissues via absorption and diffusion, they are locally metabolized and distributed systematically via the bloodstream, where they can activate the aryl hydrocarbon receptor (AhR) and promote the enzymatic generation of reactive intermediates capable of causing DNA and protein adducts [46].

The profound contribution of the dermal pathway, particularly for LMW PAHs, is strongly corroborated by human biomonitoring in both environmental and occupational settings. For instance, Lao et al. demonstrated that during exposure to combustion fumes, the dermal intake of LMW PAHs was significantly greater than the intake via inhalation, resulting in massive net excretion of urinary metabolites derived exclusively from dermal absorption [47]. Crucially, this dynamic is mirrored in heavy occupational exposures. Research on occupational PAH exposure has demonstrated that the extent of skin contamination explains the variation in the excretion of key internal dose biomarkers (such as 1-hydroxypyrene and phenanthrols) significantly better than variations in respiratory PAH concentrations [48]. These toxicokinetic mechanisms directly validate our ILCR calculations, clearly illustrating that in environments heavily contaminated with combustion byproducts and post-fire runoff, non-inhalation pathways, specifically dermal absorption, contribute critically and often predominantly to the total cancer risk.

The estimated emission, specifically PM_10_ and SO_2_, indicates a high probability of acute health effects in the immediate vicinity of the smoke plume. The emission of 1.34 t of PM_10_ is particularly concerning; epidemiological data suggest that every 10 μg/m^3^ increase in 24 h PM_10_ concentrations is associated with a 1% increase in daily mortality [23,49]. The SO_2_ flux of 441 kg further elevates the RQ, potentially exceeding short-term exposure limits and causing severe respiratory irritation [24].

The high relative uncertainty observed for PAHs (160.1%), PM_10_ (93.5%), and VOCs (114.6%) is a direct result of the stochastic nature of “cold” smoldering phases induced by firefighting interventions [5]. The application of water and synthetic foaming agent (Roteor M Premium) quenches flaming combustion but allows the internal core to continue pyrolyzing at lower temperatures [50,51,52].

This transition increases the emission factors of products of incomplete combustion (PICs) by several orders of magnitude compared to efficient flaming [24,50]. The pronounced variability in PAH emissions, expressed as a 160.1% coefficient of variation, reflects the spatial and temporal heterogeneity of the fire’s response to suppression activities. Cooling and oxygen-limiting effects at the water–foam interface may inhibit complete oxidation, thereby favoring the formation or release of organic aerosols and high-molecular-weight PAHs [5,50]. These results highlight that intensive water- or foam-based suppression can alter the distribution of environmental impacts, potentially increasing toxic air emissions, firefighting wastewater, and pyrolytic oil runoff [52,53].

## 5. Study Limitations

While this integrated source-to-receptor study provides critical insights into the toxicological legacy of tire fires, several methodological limitations must be explicitly acknowledged. First, the high epistemic uncertainty associated with the estimated emission factors—reaching over 160% for PAHs—reflects the stochastic nature of oxygen-limited smoldering and the unpredictable quenching dynamics of firefighting interventions. Consequently, the calculated pollutant mass loadings should be interpreted as robust order-of-magnitude estimates rather than precise absolute values. Second, the HYSPLIT atmospheric dispersion simulations utilized vertical layer-averaging (0–100 m AGL) and assumed a quasi-steady emission rate, which inherently simplifies the highly transient, micro-scale turbulence of an active fire plume. Third, as previously addressed, the in situ soil investigation was constrained by a highly localized sampling matrix (*N* = 5). This limits the spatial resolution of the deposition footprint and confines the multivariate statistical evaluation (PCA) to an exploratory analysis. Finally, the human health risk assessment (ILCR) relies on deterministic mathematical models to estimate potential exposure. Future research would greatly benefit from incorporating human biomonitoring data (e.g., urinary PAH metabolites) from first responders and local residents to directly validate the internal dosing pathways identified in this study.

## 6. Conclusions

The integrated source-to-receptor assessment of the landfill tire fire demonstrates that such episodic events can rapidly evolve from acute atmospheric emergencies into persistent terrestrial toxicological burdens within the surrounding environment. Quantification of source emissions indicated an inefficient combustion regime, characterized by substantial PM10 and ∑ PAH loadings. The high relative uncertainty of the organic fraction (160.1%) likely reflects the spatial and temporal variability of smoldering phases and suppression-induced changes in combustion conditions. HYSPLIT dispersion modeling, supported by concurrent concentration spikes at regional monitoring stations, indicated that plume rise and atmospheric transport contributed to a non-local deposition footprint, thereby decoupling pollutant impacts from immediate proximity to the source. This mechanism was associated with the formation of contamination hotspots in residential allotment gardens, linking industrial waste-fire events with urban food-chain vulnerability. In these areas, the cumulative ILCR for children reached 9.7 × 10^−4^, exceeding the commonly used upper regulatory benchmark of 10^−4^.

The dominance of the dermal exposure pathway, together with elevated ecological risk quotients (RQ > 1) for Zn and benzo[a]pyrene along the plume axis, indicates substantial pressure on terrestrial ecosystem health. Crucially, the identification of distal hotspots (S4) with BaP concentrations significantly exceeding international residential safety thresholds (e.g., U.S. EPA RSLs) directly links industrial waste-fire events with urban food-chain vulnerability. Given the observed disparity between MEQ and TEQ values, monitoring protocols focused solely on carcinogenic equivalency may severely underestimate the DNA-damage potential of tire-fire residues.

Consequently, we strongly recommend implementing actionable, adaptive land-use management following such disasters. This includes restricting the consumption of crops cultivated in immediate downwind sectors (such as allotment gardens) until comprehensive topsoil screening is conducted. Furthermore, long-term environmental monitoring and strict occupational health tracking (emphasizing dermal decontamination protocols) for firefighters are essential to mitigate the persistent toxicological legacy of large-scale elastomer combustion.

## Figures and Tables

**Table 1:** Estimated emissions from the landfill tire fire and associated uncertainty.

Pollutant	Emission (kg)	Uncertainty (±1 σ)	Relative Uncertainty (%)
CO_2_	4.55 × 10^4^	±2.64 × 10^4^	57.9
CO	1.12 × 10^3^	±8.40 × 10^2^	75
NO_x_	9.45 × 10^1^	±7.09 × 10^1^	75
SO_2_	4.41 × 10^2^	±3.03 × 10^2^	68.7
PM_10_	1.34 × 10^3^	±1.25 × 10^3^	93.5
CH_4_	2.52 × 10^2^	±4.03 × 10^2^	160.1
VOC (total)	4.90 × 10^2^	±5.60 × 10^2^	114.6
Σ PAHs	2.19 × 10^1^	±3.52 × 10^1^	160.1

Total emissions were calculated based on Equation (1), assuming a total consumed tire volume of 450 m^3^, an effective bulk tire density of 100 kg/m^3^, and emission factors derived from established literature [5,8,9].

**Table 2:** Estimated emissions of selected PAHs and associated uncertainty.

PAH	EF Mean (mg/kg)	Emission (kg)	Uncertainty (±1 σ)	Relative Uncertainty (%)
Naphthalene	466.25	7.34	±11.76	160.1
Acenaphthylene	196.75	3.10	±4.96	
Acenaphthene	2.78	0.0437	±0.0700	
Fluorene	45.00	0.709	±1.13	
Phenanthrene	224.00	3.53	±5.65	
Anthracene	39.75	0.626	±1.00	
Fluoranthene	124.50	1.96	±3.14	
Pyrene	109.00	1.72	±2.75	
Benz[a]anthracene	34.75	0.547	±0.876	
Chrysene/Triphenylene	44.00	0.693	±1.11	
Benzo[b]fluoranthene	30.50	0.480	±0.769	
Benzo[k]fluoranthene	40.25	0.634	±1.01	
Benzo[a]pyrene	38.50	0.606	±0.971	

**Table 3:** Concentrations of trace elements and polycyclic aromatic hydrocarbons (PAHs) in soil samples (mg/kg dw) at the fire scene and surrounding sites.

Parameters	Sampling Points				
	S1	S2	S3	S4	S5
	(mg/kg dw)				
Chromium (Cr)	16.38	75.31	16.57	17.80	20.39
Cadmium (Cd)	2.70	2.38	0.55	0.60	0.55
Copper (Cu)	1017.14	863.32	42.68	26.25	23.32
Nickel (Ni)	15.96	31.38	9.65	8.75	9.19
Lead (Pb)	133.71	490.93	26.52	38.54	22.10
Zinc (Zn)	5714.29	4184.10	1160.22	349.55	216.12
Arsenic (As)	3.81	7.25	3.45	4.11	2.83
Barium (Ba)	99.05	516.04	131.08	128.04	73.75
Total Phosphorus	1379.05	1299.86	534.53	1161.33	558.00
Cobalt (Co)	260.95	42.40	125.41	6.58	5.82
Molybdenum (Mo)	1.90	12.16	1.38	1.28	1.22
Calcium (Ca	63,070.48	24,808.93	8252.76	6686.30	5504.27
Iron (Fe)	7580.95	27,894.00	10,888.12	11,137.00	10,876.68
Tin (Sn)	11.92	68.48	2.76	2.56	2.44
Magnesium (Mg)	3251.43	3179.92	2122.93	2035.85	22.94
Naphthalene	26.67	4.88	0.51	0.06	0.06
Anthracene	16.76	1.13	0.64	1.05	0.11
Chrysene	12.76	2.09	1.93	2.30	0.35
Benzo(a)anthracene	15.43	2.79	1.93	1.54	0.37
Dibenzo(a,h)anthracene	1.83	1.13	0.25	0.33	0.09
Benzo(a)pyrene	18.67	2.51	2.49	2.05	0.40
Benzo(b)fluoranthene	26.67	4.88	2.90	2.94	0.60
Benzo(k)fluoranthene	13.71	1.53	2.49	1.92	0.24
Benzo(g,h,i)perylene	12.95	1.81	0.72	3.33	0.20
Indeno(1,2,3-cd)pyrene	3.43	2.37	1.08	1.41	0.27
∑ PAHs	148.88	25.13	14.93	16.94	2.69

**Table 4:** Incremental lifetime cancer risk (ILCR) for adults and children via ingestion, dermal, and inhalation exposure pathways to soil polycyclic aromatic hydrocarbons (PAHs).

COMPOUNDS	Sampling Points									
	S1		S2		S3		S4		S5	
	ADULTS	CHILDREN	ADULTS	CHILDREN	ADULTS	CHILDREN	ADULTS	CHILDREN	ADULTS	CHILDREN
INGESTION										
Naphthalene	7.62 × 10^−6^	12.76 × 10^−6^	1.39 × 10^−6^	2.34 × 10^−6^	0.15 × 10^−6^	0.24 × 10^−6^	0.15 × 10^−6^	0.24 × 10^−6^	0.02 × 10^−6^	0.03 × 10^−6^
Anthracene	4.79 × 10^−6^	8.02 × 10^−6^	0.32 × 10^−6^	0.54 × 10^−6^	0.18 × 10^−6^	0.30 × 10^−6^	0.18 × 10^−6^	0.30 × 10^−6^	0.03 × 10^−6^	0.05 × 10^−6^
Chrysene	3.65 × 10^−6^	6.11 × 10^−6^	0.60 × 10^−6^	1.00 × 10^−6^	0.55 × 10^−6^	0.93 × 10^−6^	0.55 × 10^−6^	0.93 × 10^−6^	0.10 × 10^−6^	0.17 × 10^−6^
Benzo(a)anthracene	4.41 × 10^−6^	7.38 × 10^−6^	0.80 × 10^−6^	1.33 × 10^−6^	0.55 × 10^−6^	0.93 × 10^−6^	0.55 × 10^−6^	0.93 × 10^−6^	0.10 × 10^−6^	0.18 × 10^−6^
Dibenzo(a,h)anthracene	0.52 × 10^−6^	0.87 × 10^−6^	0.32 × 10^−6^	0.54 × 10^−6^	0.07 × 10^−6^	0.12 × 10^−6^	0.07 × 10^−6^	0.12 × 10^−6^	0.02 × 10^−6^	0.04 × 10^−6^
Benzo(a)pyrene	5.33 × 10^−6^	8.93 × 10^−6^	0.72 × 10^−6^	1.20 × 10^−6^	0.71 × 10^−6^	1.19 × 10^−6^	0.71 × 10^−6^	1.19 × 10^−6^	0.12 × 10^−6^	0.19 × 10^−6^
Benzo(b)fluoranthene	7.62 × 10^−6^	12.76 × 10^−6^	1.39 × 10^−6^	2.34 × 10^−6^	0.83 × 10^−6^	1.39 × 10^−6^	0.83 × 10^−6^	1.39 × 10^−6^	0.17 × 10^−6^	0.29 × 10^−6^
Benzo(k)fluoranthene	3.92 × 10^−6^	6.56 × 10^−6^	0.44 × 10^−6^	0.73 × 10^−6^	0.71 × 10^−6^	1.19 × 10^−6^	0.71 × 10^−6^	1.19 × 10^−6^	0.07 × 10^−6^	0.12 × 10^−6^
Benzo(g,h,i)perylene	3.70 × 10^−6^	6.20 × 10^−6^	0.52 × 10^−6^	0.87 × 10^−6^	0.21 × 10^−6^	0.34 × 10^−6^	0.21 × 10^−6^	0.34 × 10^−6^	0.06 × 10^−6^	0.09 × 10^−6^
Indeno(1,2,3-cd)pyrene	0.98 × 10^−6^	1.64 × 10^−6^	0.68 × 10^−6^	1.13 × 10^−6^	0.31 × 10^−6^	0.52 × 10^−6^	0.31 × 10^−6^	0.52 × 10^−6^	0.08 × 10^−6^	0.13 × 10^−6^
DERMAL										
Naphthalene	135.34 × 10^−6^	159.03 × 10^−6^	24.78 × 10^−6^	29.11 × 10^−6^	17.76 × 10^−6^	20.87 × 10^−6^	2.59 × 10^−6^	3.05 × 10^−6^	17.76 × 10^−6^	0.36 × 10^−6^
Anthracene	85.07 × 10^−6^	99.96 × 10^−6^	5.73 × 10^−6^	6.74 × 10^−6^	4.11 × 10^−6^	4.83 × 10^−6^	3.22 × 10^−6^	3.79 × 10^−6^	4.11 × 10^−6^	0.66 × 10^−6^
Chrysene	64.77 × 10^−6^	76.11 × 10^−6^	10.62 × 10^−6^	12.48 × 10^−6^	7.61 × 10^−6^	8.95 × 10^−6^	9.81 × 10^−6^	11.53 × 10^−6^	7.61 × 10^−6^	2.11 × 10^−6^
Benzo(a)anthracene	78.31 × 10^−6^	92.01 × 10^−6^	14.16 × 10^−6^	16.63 × 10^−6^	10.15 × 10^−6^	11.93 × 10^−6^	9.81 × 10^−6^	11.53 × 10^−6^	10.15 × 10^−6^	2.18 × 10^−6^
Dibenzo(a,h)anthracene	9.28 × 10^−6^	10.90 × 10^−6^	5.73 × 10^−6^	6.74 × 10^−6^	4.11 × 10^−6^	4.83 × 10^−6^	1.26 × 10^−6^	1.48 × 10^−6^	4.11 × 10^−6^	0.51 × 10^−6^
Benzo(a)pyrene	94.74 × 10^−6^	111.32 × 10^−6^	12.74 × 10^−6^	14.97 × 10^−6^	9.14 × 10^−6^	10.73 × 10^−6^	12.62 × 10^−6^	14.83 × 10^−6^	9.14 × 10^−6^	2.40 × 10^−6^
Benzo(b)fluoranthene	135.34 × 10^−6^	159.03 × 10^−6^	24.78 × 10^−6^	29.11 × 10^−6^	17.76 × 10^−6^	20.87 × 10^−6^	14.72 × 10^−6^	17.30 × 10^−6^	17.76 × 10^−6^	3.57 × 10^−6^
Benzo(k)fluoranthene	69.60 × 10^−6^	81.79 × 10^−6^	7.79 × 10^−6^	9.15 × 10^−6^	5.58 × 10^−6^	6.56 × 10^−6^	12.62 × 10^−6^	14.83 × 10^−6^	5.58 × 10^−6^	1.46 × 10^−6^
Benzo(g,h,i)perylene	65.74 × 10^−6^	77.24 × 10^−6^	9.20 × 10^−6^	10.81 × 10^−6^	6.60 × 10^−6^	7.75 × 10^−6^	3.65 × 10^−6^	4.28 × 10^−6^	6.60 × 10^−6^	1.17 × 10^−6^
Indeno(1,2,3-cd)pyrene	17.40 × 10^−6^	20.45 × 10^−6^	12.03 × 10^−6^	14.14 × 10^−6^	8.63 × 10^−6^	10.14 × 10^−6^	5.47 × 10^−6^	6.42 × 10^−6^	8.63 × 10^−6^	1.60 × 10^−6^
INHALATION										
Naphthalene	5.91 × 10^−6^	2.47 × 10^−6^	1.08 × 10^−6^	0.45 × 10^−6^	0.11 × 10^−6^	0.05 × 10^−6^	0.11 × 10^−6^	0.05 × 10^−6^	0.01 × 10^−6^	0.01 × 10^−6^
Anthracene	3.71 × 10^−6^	1.55 × 10^−6^	0.25 × 10^−6^	0.10 × 10^−6^	0.14 × 10^−6^	0.06 × 10^−6^	0.14 × 10^−6^	0.06 × 10^−6^	0.02 × 10^−6^	0.01 × 10^−6^
Chrysene	2.83 × 10^−6^	1.18 × 10^−6^	0.46 × 10^−6^	0.19 × 10^−6^	0.43 × 10^−6^	0.18 × 10^−6^	0.43 × 10^−6^	0.18 × 10^−6^	0.08 × 10^−6^	0.03 × 10^−6^
Benzo(a)anthracene	3.42 × 10^−6^	1.43 × 10^−6^	0.62 × 10^−6^	0.26 × 10^−6^	0.43 × 10^−6^	0.18 × 10^−6^	0.43 × 10^−6^	0.18 × 10^−6^	0.08 × 10^−6^	0.03 × 10^−6^
Dibenzo(a,h)anthracene	0.41 × 10^−6^	0.17 × 10^−6^	0.25 × 10^−6^	0.10 × 10^−6^	0.06 × 10^−6^	0.02 × 10^−6^	0.06 × 10^−6^	0.02 × 10^−6^	0.02 × 10^−6^	0.01 × 10^−6^
Benzo(a)pyrene	4.14 × 10^−6^	1.73 × 10^−6^	0.56 × 10^−6^	0.23 × 10^−6^	0.55 × 10^−6^	0.23 × 10^−6^	0.55 × 10^−6^	0.23 × 10^−6^	0.09 × 10^−6^	0.04 × 10^−6^
Benzo(b)fluoranthene	5.91 × 10^−6^	2.47 × 10^−6^	1.08 × 10^−6^	0.45 × 10^−6^	0.64 × 10^−6^	0.27 × 10^−6^	0.64 × 10^−6^	0.27 × 10^−6^	0.13 × 10^−6^	0.06 × 10^−6^
Benzo(k)fluoranthene	3.04 × 10^−6^	1.27 × 10^−6^	0.34 × 10^−6^	0.14 × 10^−6^	0.55 × 10^−6^	0.23 × 10^−6^	0.55 × 10^−6^	0.23 × 10^−6^	0.05 × 10^−6^	0.02 × 10^−6^
Benzo(g,h,i)perylene	2.87 × 10^−6^	1.20 × 10^−6^	0.40 × 10^−6^	0.17 × 10^−6^	0.16 × 10^−6^	0.07 × 10^−6^	0.16 × 10^−6^	0.07 × 10^−6^	0.04 × 10^−6^	0.02 × 10^−6^
Indeno(1,2,3-cd)pyrene	0.76 × 10^−6^	0.32 × 10^−6^	0.53 × 10^−6^	0.22 × 10^−6^	0.24 × 10^−6^	0.10 × 10^−6^	0.24 × 10^−6^	0.10 × 10^−6^	0.06 × 10^−6^	0.02 × 10^−6^
∑ exposure route	0.00083 × 10^−6^	0.00097 × 10^−6^	0.00014 × 10^−6^	0.00016 × 10^−6^	0.00010 × 10^−6^	0.00012 × 10^−6^	0.00008 × 10^−6^	0.00010 × 10^−6^	0.00009 × 10^−6^	0.00002 × 10^−6^

**Table 5:** Ecological risk quotients (RQ) for trace elements and polycyclic aromatic hydrocarbons (PAHs) in soil samples based on Maximum Permissible Concentrations (MPC) and Negligible Concentrations (NCs).

COMPOUNDS	Sampling Points									
	S1		S2		S3		S4		S5	
	RQ_(MPC)_	RQ_(NCs)_	RQ_(MPC)_	RQ_(NCs)_	RQ_(MPC)_	RQ_(NCs)_	RQ_(MPC)_	RQ_(NCs)_	RQ_(MPC)_	RQ_(NCs)_
Chromium (Cr)	0.01	0.86	0.05	5.40	0.01	1.20	0.08	8.35	0.07	6.95
Arsenic (As)	0.02	2.00	0.05	5.20	0.03	2.50	0.09	9.28	0.13	12.84
Barium (Ba)	0.03	3.47	0.25	24.67	0.06	6.33	0.15	15.10	0.25	25.00
Tin (Sn)	0.02	1.79	0.14	14.03	0.01	0.57	0.10	10.00	0.10	10.00
Zinc (Zn)	1.50	150.00	1.50	150.00	0.42	42.00	0.35	35.40	0.55	54.60
Cadmium (Cd)	0.09	9.47	0.11	11.40	0.03	2.67	0.22	22.35	0.24	23.55
Cobalt (Co)	0.69	68.50	0.15	15.20	0.45	45.40	0.10	9.54	0.10	10.28
Copper (Cu)	0.89	89.00	1.03	103.17	0.05	5.15	0.10	9.55	0.10	10.25
Molybdenum (Mo)	0.00	0.40	0.03	3.49	0.00	0.40	0.02	2.00	0.02	2.00
Nickel (Ni)	0.02	1.68	0.05	4.50	0.01	1.40	0.05	5.02	0.05	4.55
Lead (Pb)	0.12	11.70	0.59	58.67	0.03	3.20	0.09	9.05	0.15	15.05
RQ Σ_Elements_	3.39	338.86	3.96	395.72	1.11	110.82	1.36	135.64	1.75	175.07
Naphthalene	0.88	87.50	0.22	21.88	0.02	2.31	0.05	5.00	0.05	5.00
Anthracene	0.44	44.00	0.04	4.05	0.02	2.30	0.09	9.00	0.82	82.00
Chrysene	0.34	33.50	0.08	7.50	0.07	7.00	0.29	29.00	1.80	180.00
Benzo(a)anthracene	0.41	40.50	0.10	10.00	0.07	7.00	0.30	30.00	1.20	120.00
Dibenzo(a,h)anthracene	0.30	30.00	0.25	25.31	0.06	5.63	0.35	35.00	1.30	130.00
Benzo(a)pyrene	3.06	306.25	0.56	56.25	0.56	56.25	1.65	165.00	8.00	800.00
Benzo(b)fluoranthene	0.70	70.00	0.18	17.50	0.11	10.50	0.49	49.00	2.30	230.00
Benzo(k)fluoranthene	0.36	36.00	0.06	5.50	0.09	9.00	0.20	20.00	1.50	150.00
Benzo(g,h,i)perylene	0.34	34.00	0.07	6.50	0.03	2.60	0.16	16.00	2.60	260.00
Indeno(1,2,3-cd)pyrene	0.09	9.00	0.09	8.50	0.04	3.90	0.22	22.00	1.10	110.00
RQ Σ_PAHs_	6.91	690.75	1.63	162.99	1.06	106.49	3.80	380.00	20.67	2067.00

## Data Availability

The original contributions presented in this study are included in the article. Further inquiries can be directed to the corresponding author.

## Supplementary Materials

The following supporting information can be downloaded at: https://www.mdpi.com/article/10.3390/toxics14070543/s1, Text S1: Key HYSPLIT model configuration parameters used in the forward trajectory simulation of the landfill tire fire plume.

## Abbreviations

BaP	Benzo(a)pyrene
CSF	Cancer Slope Factor
EF	Emission Factor
HMW	High-Molecular-Weight
ILCR	Incremental Lifetime Cancer Risk
LMW	Low-Molecular-Weight
MEQ	Mutagenic Equivalency Quotient
PAHs	Polycyclic Aromatic Hydrocarbons
PM	Particulate Matter
RQ	Risk Quotient
TEQ	Toxic Equivalency Quotient
