# Supplementary material for: Toxicological Legacy of Polycyclic Aromatic Hydrocarbons from a Tire Fire-Urban Soil Contamination and Cancer Risk Assessment
Source: Toxics. 2026 Jun 23;14(7):543. doi: 10.3390/toxics14070543 (PMC13431326; doi:10.3390/toxics14070543)
Supplement: Supplementary file 1 [file toxics-14-00543-s001.zip › toxics-4369190-supplementary.pdf]

toxics

Toxicological Legacy of Polycyclic Aromatic Hydrocarbons from a Tire Fire-Urban Soil Contamination and Cancer Risk Assessment

Kamil Pająk [1], Alicja Trawińska [1], Marcin Łapicz [2] and Andrzej R. Reindl [1,*]

[1] Department of Environmental Toxicology, Faculty of Health Sciences, Medical University of Gdansk, 80-210 Gdansk, Poland; kamil.pajak@gumed.edu.pl (K.P.); alicja.trawinska@gumed.edu.pl (A.T.)
[2] Department of Rescue Operations Management, Firefighting and Communication, Faculty of Safety Engineering and Civil Protection, Fire University, 52/54 Slowackiego St., 01-629 Warsaw, Poland; mlapicz@apoz.edu.pl
* Correspondence: andrzej.reindl@gumed.edu.pl

Test S1: Key HYSPLIT model configuration parameters used in the forward trajectory simulation of the landfill tire fire plume.

The fire broke out on October 13, 2025, at approximately 02:50 a.m., with the official emergency notification recorded at 02:55 a.m. Suppression operations lasted 9 h and 27 min. During the intervention, approximately 60 m^3 of water and 1300 L of a synthetic foaming agent (Roteor M Premium) were applied. The fire affected an estimated area of 450 m^2, with a total combustion volume of approximately 450 m^3.

Meteorology: Archived GDAS1
Source Location: Lat: 52.388310 Lon: 17.019689
Archive File: gdas1.oct25.w2
Trajectory direction: Forward
Level 1 height: 0m
Level 2 height: 100m
Height of the Fire: 1m,
Area of the Fire: 450 m^2,
Volume of the Fire: 450 m^3,
Duration: 567 minutes.

Total emissions (Ex) were estimated following the methodological framework of Bihałowicz et al. (2021), expressed as:

$$EM_x^i = q_y \cdot V_i \cdot EF_{\{x,y\}} \qquad \text{(S1)}$$

where q_y denotes the bulk density of the waste tires (kg/m^3) accounting for interstitial air voids, V is the estimated volume of consumed fuel (m^3), and $EF_{x,y}$ is the substance-specific emission factor (g/kg). Due to the

heterogeneous nature of tire landfill fires, characterized by oxygen-limited regimes and the suppressive effects of water/foam, emissions were temporally averaged assuming a quasi-steady combustion phase.

Table S1. Key HYSPLIT model configuration parameters used in the forward trajectory simulation of the landfill tire fire plume.

Parameter	Value	Description
Model version	HYSPLIT v5.4.2a	Last revision: 29 Jan 2026
Meteorological input	GDAS1 (1° × 1°)	Archive: gdas1.oct25.w2
Start time	02:00 UTC, 13 Oct 2025	Fire ignition: 02:55 local (CET = UTC+1)
Trajectory direction	Forward	Source-to-receptor transport
Release levels	10, 50, 100 m AGL	Near-surface human exposure layer
New trajectory interval	1 h	10 trajectories over fire duration
Simulation duration	24 h	Covers full transport and deposition window
Vertical motion	Model vertical velocity	KMSL = 0; WVERT = T
Plume rise	Briggs algorithm	PLRISE = 1; Heff = 236 m AGL
Heat release rate Q	42.3 MW	Derived from V, ϱtire, HV, t (Eq. 7)
Buoyancy flux Fb	371.8 m^4 s^{-3}	Calculated via Eq. 6
Final plume rise ΔH	235 m	Briggs formula (Eqs. 4–5)
Boundary-layer scheme	Beljaars–Holtslag	KBLT = 1
Stability derivation	Surface flux method	KBLS = 1

Time-step ratio	0.75	TRATIO = 0.75 (Courant stability)
Mixed-layer depth output	Active	TM_MIXD = 1; diagnosed along trajectory
Ambient temperature output	Active	TM_TAMB = 1; from GDAS1
Stability class	D (near-neutral)	Nocturnal, $u \approx 3.5$ m s^{-1}
READY Job ID	195793	NOAA ARL server, run 01 Jun 2026

Disclaimer/Publisher's Note: The statements, opinions and data contained in all publications are solely those of the individual author(s) and contributor(s) and not of MDPI and/or the editor(s). MDPI and/or the editor(s) disclaim responsibility for any injury to people or property resulting from any ideas, methods, instructions or products referred to in the content.
